# Fatal Septicaemia Following Suprapubic Cystostomy in a Paraplegic Patient: Never Do a Cystostomy without Prior Urine Culture and Appropriate Antibiogram!

**DOI:** 10.1155/2010/461514

**Published:** 2010-06-10

**Authors:** Subramanian Vaidyanathan, Bakul M. Soni, Tun Oo, Peter L. Hughes, Gurpreet Singh

**Affiliations:** ^1^Regional Spinal Injuries Centre, District General Hospital, Southport, Merseyside PR8 6PN, UK; ^2^Department of Radiology, District General Hospital, Southport, Merseyside PR8 6PN, UK; ^3^Department of Urology, District General Hospital, Southport, Merseyside PR8 6PN, UK

## Abstract

Neuropathic urinary bladder is often colonised by multidrug-resistant bacteria. We report a 64-year-old male spinal cord injury patient with paraplegia, who received gentamicin on empirical basis before undergoing suprapubic cystostomy, as antibiotic sensitivity report of urine was not available. This patient developed fulminate septicaemia. Although appropriate antibiotic therapy (meropenem) was started when this patient manifested features of sepsis, acute renal failure occurred and he expired. Inappropriate initial antimicrobial therapy was the major contributory factor for this patient's mortality. Learning points from this case are (1) never do a cystostomy without prior urine culture and appropriate antibiogram; (2) in a chronic spinal cord injury patient, full blood count, liver function tests, albumin level, and albumin to globulin ratio should be performed before any surgical procedure.

## 1. Introduction

We report a spinal cord injury patient in whom suprapubic cystostomy was performed without prior urine culture and appropriate antibiogram. This patient received gentamicin on empirical basis but developed fulminate septicaemia and expired within forty-eight hours. Decreased natural and adaptive immune responses have been reported in patients with spinal cord injury [1–3]. However, it remains unclear if a reduced immune response, which is pronounced in the acute phase after spinal cord injury, plays a relevant role in this patient seven years after spinal cord injury.

## 2. Case Presentation

A British Caucasian male patient, born in 1946, fell down from a ladder in 2002 and sustained concussional brain injury, soft tissue injury to left shoulder, burst fracture of thoracic vertebrae 4, 5, and 6, paraplegia T-5 (American Spinal Injury Association grade B), multiple rib fractures and mediastinal haematoma. Following rehabilitation, this patient had been managing his bladder by long-term indwelling catheter drainage.

In June 2009, he developed temperature of 38.8 degrees Celsius. White cell count was high (18.1 × 10^9^/L). Neutrophils were increased (15.85 × 10^9^/L). C-reactive protein was elevated (121.1 mg/L). Total protein: 65 g/L. Albumin: 39 g/L. Globulin: 26 g/L. Urine microbiology showed growth of *Klebsiella pneumoniae,* which produced an extended spectrum beta-lactamase and multiresistant. This patient was prescribed gentamicin 320 mg once daily. In October 2009, this patient again developed urine infection. C-reactive protein was high at 165.8 mg/L. Total protein: 69 g/L. Albumin: 38 g/L. Globulin: 31 g/L. Haemoglobin was 12.7 g/dL. Urine showed growth of Escherichia coli; antibiotic sensitivity report was not available. This patient was prescribed meropenem, one gram intravenously every eight hours. While the patient received meropenem, another sample of urine was sent for microbiology; this sample of urine showed no growth. Intravenous urography was performed in November 2009 to exclude stones in the upper urinary tract as a reason for the recurrent urinary tract infection. No renal or ureteric calculi were found.

In January 2010, this patient decided to undergo suprapubic cystostomy so that he could discard permanent catheter in the penis. At the time of undergoing suprapubic cystostomy, this patient was feeling well; he did not have symptomatic urine infection. He did not suffer from any comorbid condition such as diabetes mellitus, pressure sore, ischaemic heart disease, hypertension, or mental health problem. This patient had Medtronic programmable pump implanted for intrathecal administration of baclofen for control of spasticity. His medications were senna syrup, sugar-free, 30 mg on alternate evenings; Micralax Micro-enema 5 mL on alternate mornings; oxybutynin modified release 10 mg, once daily. Neither blood tests nor urodynamics were performed prior to suprapubic cystostomy. He was given gentamicin 240 mg intravenously, as antibiotic sensitivity report of urine sample was not available to physicians treating this patient. Suprapubic cystostomy was performed in the outpatient department using Add a Cath. The urinary bladder was located by ultrasound scan. The procedure was carried out uneventfully. Following suprapubic cystostomy, there was mild haematuria. Two hours later, this patient was feeling unwell; he had tachycardia, spasms, and cold fingers. Temperature was 38 degrees Celsius; oxygen saturation was 97%; urine was stained with blood; there was some oozing of blood from suprapubic cystostomy site. Suprapubic catheter was patent. Diagnosis was septicaemia. He had received one dose of gentamicin 240 mg intravenously. Subsequently, a review of laboratory reports revealed that in October 2009, urine showed growth of coliforms, sensitive to meropenem. Therefore, this patient was prescribed meropenem 1 gram intravenously every eight hours. He was given fluids intravenously. A Foley catheter was inserted per urethra. Blood culture was taken. This blood culture yielded growth of *Escherichia coli;* however, antibiotic sensitivity was not available. Full blood count revealed low white cell count of 2.7 × 10^9^/L, which indicated severe sepsis. Blood test showed Total Protein: 72 g/L; Albumin: 40 g/L; Globulin: 32 g/L.

Sixteen hours after undergoing suprapubic cystostomy, this patient was afebrile. He was breathing spontaneously with 40% oxygen by nasal mask. Heart rate was 93 per minute; Blood pressure was 80/33 mm Hg; respiratory rate was 16 per minute. There was peripheral cyanosis; capillary circulation return time was greater than two seconds. Examination of chest revealed bilateral equal air entry and vesicular breath sounds. Abdomen was soft and bowel sounds were present. Clinical impression was septicaemia and hypovolaemia. Rate of administration of intravenous fluids was increased to 160 mL per hour. Arterial blood gas showed pH: 7.393;  pCO2: 5.87 kPa; pO2: 8.25 kPa; actual bicarbonate: 26.2 mmol/L; standard bicarbonate: 25.2 mmol/L; base excess: 1.0 mmol/L. Full blood count showed leucocytosis (15.6 × 10^9^/L); neutrophils count was high (15.3 × 10^9^/L). APTT ratio was high at 1.40 (reference range: 0.84–1.16). C-reactive protein was high at 123.1 mg/L. Blood urea: 9.1 mmol/L; creatinine: 148 micromol/L; sodium: 133 mmol/L; potassium: 4.3 mmol/L.

Thirty-four hours after performing suprapubic cystostomy, this patient was found to be confused. Heart rate was 113 per minute; blood pressure was 119/63 mm Hg. Physical examination revealed clear chest; abdomen was silent; penis was oedematous. Blood tests showed sodium: 133 mmol/L; potassium: 4.3 mmol/L; urea: 9.1 mmol/L; creatinine 148 micromol/L. This patient was given intravenous infusion of succinylated gelatine (Gelofusine) 500 mL. Even after this fluid challenge, urine output was only 15 mL. Both urinary catheters were patent. On clinical examination, abdomen was soft and nontender. Urinary bladder was not palpable; suprapubic wound was healthy; penis was very oedematous.

Thirty-six hours after undergoing suprapubic cystostomy, this patient was in renal failure; urine output was 5 mL in two hours. Full blood count showed very high white cell count (23.6 × 10^9^/L). Haemoglobin was slightly low at 10.5 g/dL. Platelet count was also low at 12 × 10^9^/L 1. Neutrophils were greatly increased (22.4 × 10^9^/L). Blood urea had risen to 16.4 mmol/L. Creatinine concentration also had increased to 253 micromol/L. Sodium was low at 127 mmol/L. Potassium level had increased from 4.3 mmol/L to 5.4 mmol/L. This patient developed cardiac arrest 38 hours after suprapubic cystostomy. After 28 minutes of unsuccessful resuscitation, he was declared dead. Patient's wife did not give permission for autopsy.

## 3. Discussion

Iatrogenic bowel injury is a recognized complication of percutaneous suprapubic cystostomy [4]. For safe placement of a suprapubic catheter, the patients must have an adequately distended bladder and be placed in Trendelenburg position that allows safe extraperitoneal puncture of the bladder. In this patient, suprapubic cystostomy was performed under ultrasound guidance after filling the bladder with 0.9% sodium chloride through urethral catheter. When this patient developed septicaemia, there were no clinical features of peritonitis such as abdominal distension, guarding, rigidity, or rebound tenderness. In spinal cord injury patients, normal clinical signs for peritonitis can be absent due to sensory deficits. Therefore, computed tomography of abdomen should be performed in spinal cord injury patients to rule out intraabdominal pathology such as bowel perforation or extravasation of urine. We should have carried out autopsy to ascertain the precise cause of death especially in this patient, who died within 48 hours after undergoing a surgical procedure.

Spinal cord injury produces profound alterations in lower urinary tract function such as incontinence, elevated intravesical pressure, vesicoureteric reflux, renal stones, vesical calculi, and detrusor-sphincter dyssynergia, which increase the risk of urinary infection in these patients [5]. Further, colonisation of neuropathic urinary bladder by multidrug-resistant bacteria is not uncommon in spinal cord injury patients. In a study of 145 patients suffering from spinal cord injuries, admitted to the Institute for physical medicine and rehabilitation, Centre for paraplegia of the Clinical Centre of the University of Sarajevo [6], a total of 4539 urine samples were obtained for microbiology. Of these urine samples, 3963 (87.3%) were positive and 576 (12.7%) were sterile. 55.3% of isolates were multi-drug resistant, and the highest rates of resistance were found among *Acinetobacter baumannii (*87.8%), *Providencia rettgeri* (86.7%), *Pseudomonas aeruginosa* (85.4%), *Providencia stuarti* (84.3%), and *Morganella morganii* (81.0%). 

Waites et al. [7] studied urine samples from two hundred eighty-seven patients with spinal cord injury, who attended clinics. There were 706 gram-negative isolates from 444 urine specimens. Occurrence of bacteriuria with gram-negative organisms demonstrating resistance to antimicrobial agents in 2 or more classes was observed in 33% of bacterial isolates.

In spinal cord injury patients, antibiotic therapy is only indicated in symptomatic bacteriuria or in symptomatic exacerbations of chronic urine infection [8]. A study of 38 patients with spinal cord injury, who underwent 51 urologic procedures between January 2004 and June 2005, revealed that twenty-six patients needed antimicrobials other than gentamicin (piperacillin/tazobactam, cefotaxime, ceftazidime, imipenem/cilastatin, ciprofloxacin, co-amoxiclav, or amikacin) because uropathogens were resistant to gentamicin [9]. These pathogens consisted of *Pseudomonas aeruginosa *(7 isolates), *Escherichia coli *(6 isolates), *Klebsiella *species (10 isolates), *Serratia marcescens *(2 isolates), *Enterobacter *species (2 isolates), and *Staphylococcus aureus *(1 isolate).

Presence of multidrug-resistant bacteria in urine of spinal cord injury patients warrants administration of *appropriate *antibiotic(s) before performing any urological procedure and continued for 24–48 hours. If a spinal cord injury patient receives antibiotics to which urinary bacteria are resistant, such a patient may develop bacteraemia and septicaemia following an invasive urological procedure, as indeed happened to our patient.

Kumar et al. [10] showed that the inappropriateness of initial antimicrobial therapy remained most highly associated with risk of death in patients with septicaemia, as exemplified by this case. Delays to appropriate antimicrobial therapy have been shown to contribute to significant increases in the incidence of acute kidney injury [11]. Our patient, who was given gentamicin on empirical basis developed septicaemia and renal failure after uneventful suprapubic cystostomy. He developed cardiac arrest before we could organise haemofiltration.

We did not perform blood tests prior to suprapubic cystostomy. In hindsight, we realise our mistake. In a chronic spinal cord injury patient, full blood count, liver function tests, albumin level, and albumin to globulin ratio should be performed before any surgical procedure. If blood tests show any nutritional deficit, such deficit should be corrected before performing any surgical procedure. Correction of nutritional deficiency is even more important than dual antibiotic therapy in a chronically ill patient.

## 4. Conclusion

1. A sample of urine should be sent for microbiology in all spinal cord injury patients prior to major urological procedures such as suprapubic cystostomy, removal of stones from urinary tract, insertion of urinary stents, nephrostomy, urethral sphincterotomy, bladder neck resection, and urethrotomy. *Antibiotic sensitivity report of urine should be available to physicians, who treat spinal cord injury patients. *
2. Appropriate antibiotic(s) should be prescribed in adequate doses on the basis of recent urine microbiologic test results immediately before performing urological procedure and continued for 24–48 hours. *Never do a cystostomy without prior urine culture and appropriate antibiogram! *
3. If antibiotic sensitivity report of recent urine sample is not available, dual antimicrobial therapy consisting of an aminoglycoside (gentamicin or amikacin), and either carbapenem (imipenem with cilastatin or meropenem) or antipseudomonal penicillin (piperacillin with tazobactam) should be given to spinal cord injury patients undergoing urological procedures until microbiology report is available.
4. If a spinal cord injury patient develops features of sepsis after undergoing suprapubic cystostomy, computed tomography of abdomen should be performed to look for silent bowel perforation or extravasation of urine, as normal clinical signs for peritonitis can be absent due to sensory deficits.
5. Long-term urethral catheter drainage is not the ideal method of managing neuropathic bladder in spinal cord injury patients. All efforts should be made to discard indwelling urinary catheter and start intermittent catheterisation regime. Never leave a patient on Foley catheter for seven years!
